# Maker Cultures and the Prospects for Technological Action

**DOI:** 10.1007/s11948-016-9796-8

**Published:** 2016-07-07

**Authors:** Susana Nascimento, Alexandre Pólvora

**Affiliations:** 1 European Commission - Joint Research Centre (JRC), CDMA 04/027 Rue du Champ de Mars 21, 1050 Brussels, Belgium; 2 European Commission - Joint Research Centre (JRC), CDMA 04/044 Rue du Champ de Mars 21, 1050 Brussels, Belgium

**Keywords:** Maker culture, Do-It-Yourself, Empowerment, Technological action, Makerspace, Fab Lab

## Abstract

Supported by easier and cheaper access to tools and expanding communities, maker cultures are pointing towards the ideas of (almost) everyone designing, creating, producing and distributing renewed, new and improved products, machines, things or artefacts. A careful analysis of the assumptions and challenges of maker cultures emphasizes the relevance of what may be called technological action, that is, active and critical interventions regarding the purposes and applications of technologies within ordinary lives, thus countering the deterministic trends of current directions of technology. In such transformative potential, we will explore a set of elements what is and could be technological action through snapshots of maker cultures based on the empirical research conducted in three particular contexts: the Fab Lab Network, Maker Media core outputs and initiatives such as Maker Faires, and the Open Source Hardware Association (OSHWA). Elements such as control and empowerment through material engagement, openness and sharing, and social, cultural, political and ethical values of the common good in topics such as diversity, sustainability and transparency, are critically analysed.

## Introduction

We are witnessing a rise in new Do-It-Yourself (DIY), crafting, manufacturing, hacking, fabbing, or making paradigms where a mix of tools, communities and spaces are increasingly enabling more and more people to produce and share knowledge at a quicker pace, create their own material and symbolic solutions, and define the goals and outcomes of their technological actions. Professional and amateur inventors, crafters, hackers, entrepreneurs, artists, scientists, engineers, designers, teachers, or activists, from nearly all ages and backgrounds, are currently not only thinking about how to transform their material environments, but also taking their own steps in that direction by learning how and choosing to modify, assemble, create, disassemble, recreate, duplicate, and sharing objects and systems through open and collaborative networks from their homes, garages, schools, businesses, museums, libraries, makerspaces, hackerspaces, Fab Labs, and other emerging innovation-oriented spaces for which we still need designations.

This movement is taking place because a wide group of people is increasingly acquainted with a growing and diversified cheaper set of tools and machines intended for personal manufacturing (Mota 2011), such as digital fabrication devices (CNC machines, 3D printers, laser cutters, etc.), open source and low-cost hardware (Arduino, Raspberry Pi, etc.), and all the multiple digital and analogue add-ons they are able to link to these devices, from the newest ambient sensors to the oldest plywood shapers. But this is also happening because the same social actors are choosing to engage with technology while sharing most of their work through documentation and data repositories, supporting others through tutorials or financial and logistical backing, and most important, collaborating on widely diversified platforms also with varied levels of engagement and openness, by organizing online and physically permanent and non-permanent meetups and workshops, and subsequently establishing peer-production communities (Troxler 2010).

Broadly called makers, these heterogeneous communities are now seen as the vanguard agents in creating, experimenting, producing and distributing new technological solutions, and as such, leaders in generating disruptive innovations that largely affect scientific, economical, educational or government organizations, and ultimately, societal structures as a whole (Deloitte 2014). Its relevance became apparent in recent years even at higher policy levels throughout the world following increasing support for multiple initiatives and discussions. We can observe it for example in the USA with President Obama talking about “the promise of being the makers of things and not just the consumers of things”^1^ in the 2009 Campaign “Education to Innovate”, or more recently in 2014, when he proclaimed June 18 as the National Day of Making at the White House Maker Faire, committing it to the “democratization of technology”^2^ in the presence of amateurs from all ages among scientists and high officials. We can also note it in the growing political weight of these trends on our side of the ocean, with their acknowledgement in European Commission events, as the Workshop “Future Horizon 2020 R&I Challenges and Opportunities” hosted in 2014 by DG CNECT, namely with debates around “The prospects of a ‘Do-It-Yourself’ innovation ecosystem”,^3^ or yet in the discourse of political agents, with former Commissioner Neelie Kroes stating the benefits of the open paradigm, in her public address at the 2014 Open Knowledge Festival: “the more you share ideas– the more others can build on them. It’s a new way of operating and thinking. (…) Information can long sit in dusty drawers– but it only gains value when opened up.” (Kroes 2014). And we can equally see it in other parts of the world, from the creation of the Digital Culture Points Network aimed at grassroots citizen engagements with media and hacking cultures, all over Brazil since 2004,^4^ to the awareness that has been triggered by sprouting collaborative support structures and gathering at events by Maker Faire Africa since 2009, in cities as Accra, Cairo, Nairobi, Lagos, and Johannesburg.^5^

A broad set of questions must emerge, nonetheless, in the midst of these transformations, on how this seemingly new availability of tools and contexts to design, modify, create and distribute objects and systems is really pointing to expanded social autonomies regarding the purposes and applications of these same objects and systems within our ordinary lives. It is usual that we tend to assemble different realities of making in the same group, thus encompassing a broad scope of people or communities with miscellaneous objectives under the same umbrella, often regardless of their dispositions towards creative expression or technological curiosity, commercial goals or ethical commitments and political intentions. But an understanding of the societal impact of makers requires a sounder framing of the principles, values and practices that compose maker cultures. And above all it requires understanding of how differences within them may reflect various levels when countering technological determinism, here not necessarily in its “hard” version where technological development follows a fixed and autonomous path of growth and perfectibility, but a “softer” one in which technologies and their structures permeate and steer social and political features of society (Marx and Smith 1994).

By examining the assumptions and challenges of these groups in the spheres of societal change, we argue for different perceptions about them and their leading contexts. We position their interventions as a social whole within the large sphere of technological actions, but we chose to review them looking for active and critical interventions of citizens in the actual design and building of technologies with the material and symbolic chances offered by these maker cultures, in heuristic opposition to the passive and often uncritical interventions that constitute our main ways of dealing with the macro and microscopic technical realms in which we all live. The perspective we will present sees most of these maker interventions entailing not only their most visible and debated technical features, but above all a complex and interconnected play of social, cultural, ethical and political elements, which fully deserve critical reviews as possible alternatives to the many of the deterministic trends that seem to dominate current major technical pathways of invention, production, distribution, use, and even discard.

An introductory section starts with a reflection on affiliations, engagements and critical possibilities that comprise the diversified background of the current realities of making, while introducing the idea of active and critical interventions in the wider realm of technological actions as a possible framework to integrate the elements of makers material and immaterial relationships with technology. The following section and the remainder of the paper presents snapshots of maker cultures based on empirical research conducted in three particular institutional maker contexts: the Fab Lab Network, Maker Media core outputs and initiatives such as Maker Faires, and the Open Source Hardware Association (OSHWA). It is divided into 3 subsections which explore a set of elements that could be technological action in maker cultures for expanded social autonomies able to counter technological determinism: the first on the question of control and empowerment through material engagement; the second on openness and sharing; and the third on social, economic, cultural, political and ethical standards and values of common good in topics such as diversity, sustainability and transparency.

## Making Between Affiliations, Engagements and Critical Possibilities

In the most commonly used definitions, makers are those who tinker, fix, recreate or assemble objects and systems in creative and innovative directions, commonly adhering to the search for alternative and non-deterministic pathways to live in contemporary material worlds. Their profile has been mostly popularised in recent years by outfits such as Maker Media, which offers DIY electronics, tools, kits, and books through its online and pop-up Maker Shed stores, but also publishes MAKE, a bimonthly magazine showing step-by-step DIY and maker projects, and produces Maker Faires all around the world as DIY festivals of science, arts and crafts. Within Maker Media’s outputs terminology, the maker movement appears as a social collective strongly oriented towards the values of technical creativity and self-expression, openness and knowledge sharing, community building, and alternative innovation (Anderson 2012; Hatch 2014). Their public message is that anyone can and should have access to tools and communities to build or rebuild anything they might want or need, standing out as a self-empowering vision of present worlds where consumers should have all necessary skills to become producers or creators.

At the first level, DIY itself can be seen in the background of current maker trends as a social phenomenon broadly linked to activities of creation, modification or repair of artefacts without professional or expert assistance. This was originally tied to hobbyist practices dealing with various issues, from motorized vehicles and home improvements to amateur inventions or semi commercial ventures, but it was also constantly intertwined with a defence of intrinsic values in self-production or wider protests against consumerism (McKay 1996; Spencer 2008; Kuznetsov and Paulos 2010). DIY contexts always allowed people from diverse backgrounds their own ways to open up the black boxes of technology, while nurturing a shared understanding of hands-on interventions, adding social or political values to their productions, and contributing to what is now understood as the maker ethos. However, we need to look into other contexts such as the original ones of hacking, in order to better understand maker cultures as having a potential to epitomize less passive technological actions while opposing more deterministic models.

In praise of “making is connecting” (Gauntlett 2011) with things, people or the world, or making something as an act that implies a different type of relationship with one’s environment, has always been present in hacker contexts. Steven Levy’s (2010) seminal analysis about hackers points us exactly in this direction where the act of hacking in itself implies changing the worlds one has as surroundings. One of the main features he spotted in hacking was a “Hands-On Imperative” dictating how essential knowledge about the world came from taking things apart, understanding how they work, and creating new and more interesting ones. And it is by following this path that we start connecting both hacker and maker cultures on an attitude, based in solving problems and building things through freedom of action and voluntary mutual assistance, particularly translated in hacking by common values such as a hacker ethic, composed by two normative principles presented in the Hacker Jargon File (a repository of computer programmer slang started in the 70 s, now in its version 4.4.7): the act of sharing as a “powerful positive good” coupled with system-cracking as ethically acceptable in the absence of theft, vandalism or breach of confidentiality (Raymond 1996).

If hacker worlds always had a specific mix of ideals of freedom, access, transparency, equal opportunity and social good, which are now possible to detect in the largest part of maker contexts the relation between these two cultures has also more convoluted aspects. Framed by disseminated beliefs that “hackers should be judged by their hacking, not bogus criteria such as degrees, age, race, or position” (Levy 2010: 31), technology was mostly assumed in hacking spheres as a social equalizer and adjuster of arbitrary differences that undermines the individual and collective potential based on meritocratic systems. Even if some prefer to focus particular innovation benefits brought by such levelling features to technical systems in which the most able or talented persons are to be socially or economically rewarded due to perceived abilities or achievements, we can also effortlessly connect hacking to making through this same point with inclusive ideas of improvement for all, founded on the notion that anyone who intends to should benefit from the power of technology through productive and creative ways of hacking (idem: 37).

The latter dynamics are at the general core of making cultures as we find their values and practices heavily inscribed in most maker acts and discourses. But as it happened, and still keeps on happening in the larger part of the hacking movement, we can’t connect it to making without realising that we still fail to understand how these dimensions oriented towards equal access are strongly informed and conditioned by social and cultural features around and within technologies themselves, such as disparities in social, cultural and economic capital, gender and ethnicity, or geographical centrality. We still tend to neglect how the meritocratic levelling of the playing field rarely matches a levelling of opportunities to access that same field and keep on engaging with it. Problems like these may not be ignored as obstacles to the potential of maker cultures to counter deterministic models in several levels of the technology field. They generally end up being subsumed by perspectives where the focus in making belongs to goals such as attaining higher levels of technical or intellectual ability, under a plain mastering of tools and information that allow taking things apart, studying their constitution, the creation of new or recreation of old ones.

Having effectively grown and spread from cultures such as hacking, maker cultures apparently incorporate not only the ultimate ideals of liberation and unlimited empowering action through technology, but also a complex relationship with more socially or collectively aware values and practices that makes it almost impossible to understand the latter without observing the former. The often simplified quest for technical control was and still is a powerful force in hacking, favouring both the pursuit of “quality and excellence in technical production” (Coleman 2014), and an aesthetic sense as something joyful within an intrinsically gripping finale (Himanen 2001). It is also a force in making that is becoming increasingly omnipresent. Maker engagements with the world can easily embrace a sense of freedom and creativity to make whatever is wanted, or as rewarding in itself as a manifestation of one’s abilities, with no major calls for changes in this situation, or even no concrete attention to its social conditions and consequences.

It is here that although we find a need to recognize how alternative active interventions of citizens are happening inside maker cultures by drawing from other notions in regard to critical technological actions. They also exist in some fractions of hacking cultures and entail not only the most visible technical features, but a complex play of social, cultural, ethical and political elements that they choose to deal with as technological actions. Some makers have been more prone to invest at the intersections between personal fabrication, DIY and craft practices, physical computing or open source electronics, with viewpoints on maker cultures as contexts for the empowerment of ordinary lives (Mellis et al. 2007; Buechley and Perner-Wilson Buechley and Perner-Wilson 2012). More interventionist views on making as the possibility of control over technology or even defiance of larger technological structures heavily oriented by deterministic principles started to emerge, when it came to issues such as practicability of everyday use, access to components, constraints of existing skills and interests, opacity of tools and devices, etc. (Mellis and Buechley 2014).

When practitioners or scholars argue within maker cultures for lay people to become active designers, producers and distributors of technologies, goods and services, something more is surely added to the large realm of technological actions (Nascimento 2014; Nascimento and Polvora 2013). It is thus fundamental to understand not only how they are able to voice and materialize their ethical, cultural or political concerns, but also how this may change the definitions of the culture they are referring to. This can have profound effects for example in parts of the heterogeneous contexts we define as maker cultures showing once more their affiliations to cultures such as the one of hacking, and putting them in line with paradigms such as that of “critical making”, seen as politically transforming maker activities (Ratto and Boler 2014), or even adding their individual or collective makers to technology-oriented and product-oriented movements (TPMs), professed as citizen mobilizations to support alternative technologies with the intent of triggering social change (Hess 2005).

A convergence in the field of Science and Technology Studies (STS) has tried to underline this sort of change in recent years when discussing the innovative agencies of users and communities in designing, building and distributing their own solutions, often in opposition to already existing centralised or tight institutionally controlled prevailing communities (Oudshoorn and Pinch 2003; Van Oost et al. 2009). Their focus was quite similar to the issues we now note in particular sections of the maker culture, which are becoming more active and more critical in the realm of their technological actions, from intense awareness to social backgrounds, current status and social diversity, to alternative normative ways towards more democratic and sustainable societies (Woodhouse and Patton 2004), or the role of beliefs or political commitments and their awareness of innovation (Söderberg 2011). And here it was made clear that when some technologically engaged groups decide to exhibit social and political orientations in their actions, the same actions may be catalysts of their larger contexts in ways that help the groups themselves express and enact even more value laden perspectives, therefore getting closer and closer to particular social and political platforms (Winner 1986; Verbeek 2005; Vermaas et al. 2008).

In the following section, we will analyse how these active and alternative possibilities to design something from scratch, to modify a certain device, or to repurpose it to other functions and ends, are to be understood within an integrated and expanded understanding of specific elements in several contexts of maker cultures, thus exploring the relevance of new forms of technological action. The promises and challenges of maker cultures now point towards new counter actions to technological determinism in science and technology, and we need to assess how tangible is the shift and what kind of impacts it may have in the next subsections elements such as control and empowerment; openness and sharing; or political and ethical questionings.

## Snapshots of Maker Cultures and Contexts

### Gaining Control and Empowerment

Through making, one’s understanding of the surrounding environment, and subsequently one’s power within it, can be transformed following developments in the skills that arise from the invention or recasting of artefacts and systems. Gaining or possessing capabilities in this context relates to the concept of social control over technology, as we can see at the centre of a discourse by Dale Dougherty, founder, President and CEO of Maker Media, stating: “you’re makers of your own world, and particularly the role that technology has in your life. (…) Makers are in control. That’s what fascinates them; that’s why they do what they do. They want to figure out how things work, they want to get access to it, and they want to control it; they want to use it to their own purpose” (Dougherty 2011). This self-empowering narrative is the key to understand the depth of change that making can impart to technological actions, where a more direct knowledge and intervention of non-experts in technologies moves from the plainest notions of humans as being “craftspeople” (Sennett 2009), to more intricate matters of social, cultural or political rights in taking part in the technological process by accessing tools and making something with them (Sclove 1995).

It was with a similar understanding that Neil Gershenfeld (2005) introduced the concept of Fab Lab at the MIT’s Center for Bits and Atoms (CBA), and started a particular network of Fab Labs towards the goal of democratizing production, that is, of allowing anyone to make (almost) anything, in spaces now seen as an “open, creative community of fabricators, artists, scientists, engineers, educators, students, amateurs, professionals, ages 5 to 75+”, which share “the goal of democratizing access to the tools for technical invention”.^6^ This network reported 413 active and planned units in November 2014 with a steady growth around the world since its constitution as “makerspaces”, probably the most broadly accepted term for “innovative workshop spaces that allow people to access tools freely and make things in collaborative projects” (Smith et al. 2014). And while some critically prefer to maintain an open field opting for non-institutionalized terms as “shared machine shops” to better describe this kind of “real-life laboratories as new places for experimental innovation practices in contexts of peer production” (Dickel et al. 2014), Fab Labs are indubitably the current main example of “makerspaces” (Bosqué et al. 2015, Menichinelli et al. 2015).

A dialogical intertwining of our insights and extended literature assessments with samples of empirical reviews on particular maker environments, gathered as part of a larger research mainly supported by qualitative inquiries, namely semi-structured interviews and participant observation, allows us to assess more thoroughly a context such as the one of Fab Labs. With an ethnographic anchor in FAB10 Barcelona (2–8 July 2014), the annual gathering of the international Fab Lab community, we conducted 9 interviews with present and former Fab Lab coordinators or managers from Germany, Italy, Netherlands, France, Austria, Denmark and Brazil. Interviewees here largely expressed how Fab Labs are powered by the desire and passion of their users to make things, that is, to solve problems, to tinker, to experiment or hack. But on a second level, however, with the motivation to bring about their projects, they also stated users’ ideas about the possibilities of ushering more social control through the acts of making, in a new type of empowerment, even defined by one interviewee as “immediate exposure to materiality”.

In these contexts we may see a sense of alteration of the world through concrete material engagement. Through enhancing the relation with surrounding spaces, more complex, direct and active relationship with technology can be developed in regard to the technical aspects of everyday life existences (Ihde 1990). In the Fab Foundation description of “What is a Fab Lab”, its participants are assumed to be “empowered by the experience of making something themselves” as “both learn and mentor each other, gaining deep knowledge about the machines, the materials, the design process, and the engineering that goes into invention and innovation”.^7^ Most interviewees at FAB10 Barcelona underlined this idea mentioning that from their positions, Fab Lab users and participants gain not only a new understanding of the operating schemes of objects, by figuring out how to fix them, or find new solutions to repurpose them, but also a new understanding of the prospects of changing their life world conditions.

Practices of repair are often the focus of workshops or special days in some Fab Labs, and can be seen as a way to cope with and to regain particular degrees of control that surpass mere technological gains and domains:

“I think that when they can repair things by themselves, they gain a feeling of empowerment, of being able to take back control and see an object and think, ‘Yes, we can have an impact or control’ certainly because it is broken, we can take it apart and see how it works or with the help of others just solder something back in or find a solution and I think that is my favourite thing about Fab Labs. (…) Maybe one of the most interesting things to do is to demystify technology” (Fab Lab interviewee)

Furthermore, a more in-depth knowledge about technologies and tools can take several paths. For instance, the experience of actually making something, including all the obstacles, delays and deviations, may lead Fab Lab users to understand the complexity of supply chains and the use of resources. And thus it can enable users to move beyond being passive consumers of packaged or commercial products, or to at least be more conscious about the production processes of our things, objects, devices or systems. On the other hand, the ability to make something from start to finish in a place such as a Fab Lab is seen as a possible enabler of users to become creators and producers, and through this process have a disruptive impact, not only on the invention and production cycles, but also on the social, cultural, political and ethical cultures in which they are inserted. As an extension of such a disruption, for example, some of the interviewees made reference to capitalist critiques of production and power dynamics, in the sense that Fab Labs may offer an alternative model for local and micro production, heavily based on challenging and changing predetermined technological realities with material and conceptual tool sets from peer-to-peer platforms, collaborative commons, FLOSS (free/libre and open source software), etc.

Additionally, Massimo Banzi, one of the founders of Arduino, has stated that at core of this open source microcontroller is the goal “to help everyday people, people with no background in electronics or software to be able to create using technology” (2014b), that is, to build devices in an easy way to solve their own practical problems or create their own social realities. His statements were made at the inaugural presentations of Maker Faire Europe in Rome (28 September–5 October 2014), a Maker Media festival of science, arts, crafts, and DIY projects gathering around 90,000 visitors with about 600 projects plus 360 workshops,^8^ and another context that we also have empirically explored through ethnographic participant observation and interviews. Banzi’s ideas strongly supported by what he previously stated on other occasions speaking about how “we can make tools that empower everyday people to become creators of technology—and not just consumers” (Banzi 2014a), which clearly points to an alternative and active relation of users to technology.

Events like these can help us to frame a visible side of maker cultures given the growing cultural and economic importance and reach of Maker Media in defining the maker movement, especially in supporting the next generations of craftspeople, tinkerers, hobbyists and inventors. Here it is discernible for example that the use of open source platforms within active technological actions based on the will to counter specific material limitations of daily life worlds. Arduino is one of the most known, discussed, used, or even appropriated platforms of this kind, and it’s the basis for multiple showcased projects in the fields of home automation or the Internet of Things (IoT), which are explicitly designed for acquiring more technical control over things. “Smartize It!”^9^ was presented at the Faire as a platform that enables anyone with a basic Arduino knowledge to create their own low cost wireless network of smart objects in their home, laboratory or small business, and also at the Faire, projects by the IoT Zurich Meetup^10^ had in their genesis a DIY/hacker rationale that imaginatively and critically explored IoT technologies, while mostly supporting a model of bottom-up innovation composed of a mix of everyday life materials and open source tools.

A general argument comes through that large communities of people from different backgrounds, skills and knowledge, and not only from conventional institutions of academia, research centres, institutes, business or industry, are able to directly intervene in technological innovation. The goals of empowering makers to create their own things and material solutions, thus contributing to the emergence of less passive or predetermined technological actions, seem to be at the core of maker cultures, with visibility at several instances in the Maker Faire and its mother organization, Maker Media, while more clearly articulated in contexts as the Fab Lab network via their founding statements and criteria.

### Observing Openness and Sharing

The possibility to pursue more active technological actions and control over one’s worlds through different engagements with technology, requires as much access as possible to tools and information, strongly linking it to beliefs of sharing and openness. At the core of maker cultures, such beliefs frame and open up at the same time possibilities for anyone to change or create things by using all the technical means available, and by connecting with others and exchanging ideas and supporting each other in the most varied online and physical groups, communities and events. In some interventionist instances of making, the realities of accessing and sharing with others can even point to stronger oppositions to deterministic schemes, which prevent the opening up or hacking of most of our technical objects, systems or machines. In this latter sense, it is impossible to disengage making from open source principles and practices, even though open source is implemented according to different interpretations from the strands of hacking to those of making. What is now a free/libre or open source ethos has its own internal divergences over meanings and purposes that mainly arose in the software world “free software” vs “open source”. Despite a few problematic debates on their own restrictions on the use of nonfree or proprietary solutions, Richard Stallman and the Free Software Foundation^11^ contend that the Open Source Definition^12^ stripped the movement of its ethical character in order to make it more user, business or marketing appealing,^13^ relying mostly on its technical superiority compared to other technology models, as described in Raymond’s (2001) influential account on the “bazaar” development model. And Maker Media is a division of O’Reilly Media which was founded by Tim O’Reilly, one of the main proponents at the Freeware Summit in 1998 to replace the term “free software” with “open source” (also having Raymond as one of its main disseminators) and present it as an efficient development model (Coleman 2013: 78–79).

Even if strongly debated in hacking cultures, this original division is somewhat diluted in the present maker narratives around the goals of openness and sharing. The term “open source” is certainly the most popular and adopted at maker contexts and most of the projects coming from young or old, expert or amateur, digital or analogical makers, end up choosing and supporting, if not always FLOSS (Free/Libre/Open Source) software and hardware, at the very least, an implicit rationale that each maker is free to choose which tools to use, how to apply those tools, and more important, how to frame and share their creations without having major restrictions. One of the basic criteria for a context as that of a Fab Lab^14^ is precisely the sharing of a common set of tools and processes in order to allow for an exchange of knowledge and designs, which leads to a recommendation of a list of machines, materials, freeware and open source software (available on CBA’s website) that should be found in a Fab Lab in all countries. Nevertheless, it stands foremost as a recommendation which can be also identified in one of the tenets of the Fab Lab Charter^15^ regarding the freedom to choose the ownership of an invention.

But although the Fab Lab Charter also states the responsibility of knowledge dissemination through documentation and instruction, there is a sporadic use of Wikis, Tumblr, or repositories for code and materials concerning the projects that are done in Fab Labs. The absence of extensive documentation was considered by one of the interviewees to be related to the physical character of making an object and its “implicit knowledge”, which in the end, is harder to translate, to document and to share than code:

“(…) In software you always remain within the world of your screen, (…) and it is very easy to document the code inside the programme, every programming language has a means to write commands into the code. Now in Fab Labs, we are always switching between the digital design and the material manufacturing and where things go wrong is in the material manufacturing. (…) this very logical and closed connection of coding and documenting is lost in this switch between ‘digitality’ and materiality.” (Fab Lab interviewee)

In this point, it is interesting to note a different approach to the principles of openness and sharing applied to the physical character of making, which stems from the Open Source Hardware Association (OSHWA), another empirical context we explored through observation and interactions with organizers and participants in the 2014 Open Hardware Summit^16^ (September 30–October 1 2014, Rome). OSHWA aims to promote and advance the use of open source hardware by organizing its community around a common set of values, principles and norms, and by fully documenting the development of the movement (Gibb 2014). The Association started in 2010 through common discussions and meetings between several scholars and practitioners (such as Mellis et al. 2007), which evolved into an Open Source Hardware Definition (currently in its version 1.0^17^) and the first Open Hardware Summit in 2010 with approximately 320 participants, followed by Summits in 2011 and 2012, with close to 350 attendees plus 22 speakers, and 500 attendees plus 42 speakers, respectively.

OSHWA’s particularity stems from their character as an organization actively discussing the standards and even certification systems that can practically frame material technologies, partially following the paths of the FLOSS community. As advocates of the open hardware community, OSHWA can be framed as a technology-oriented and product-oriented movement (TPMs), as previously defined as a mobilization of civil society to support alternative technologies or products and their associated policies with the intent of triggering social change. A considerable part of their work is centred on promoting the definition of OSHWA and advising on best practices for releasing an OSHWA product, choice of licences, and resources for companies and individuals. For this movement, the principles of openness and sharing are to be inscribed in an open source framework, which in their view better realizes a critical technological action that develops an alternative relation to closed and deterministic technical systems of production, distribution and use.

Openness and sharing, however, are not restricted to formal mechanisms for exchange of knowledge such as documentation and licences, as just discussed. Even in more crucial ways, these principles imply the creation and development of communities invested in collaborating and supporting its members and also in principle a wider public. For a fairly restricted and small community as OSHWA, such concerns were voiced for instance in a workshop of the 2014 Open Hardware Summit, particularly on a table about community access, in terms of overspread of communities in divergent paths, loss of gurus or experienced users, starting a community from scratch or maintaining motivation over time. Some of best experiences and practices point towards building smaller and decentralized communities, co-management of resources, and also having community guidelines (“moral infrastructure or values”) and a standardized language for beginners for example in centralized places or forums to find and share information.

In the Fab Lab context, despite explicit efforts to engage users and support collaboration, there is also a number of difficulties in building and maintaining strong local communities, establishing local relevance to diverse stakeholders, or even collaborating with other Fab Labs in a continued and sustained way.^18^ Nevertheless, Fab Labs show a variety of informal mechanisms for openness and sharing framed in an extended community setting. Interviewees in general acknowledged that users liked to show their projects to others, and sharing happens informally through random encounters or exchanges of ideas, fuelled by curiosity about others’ on-going projects. More importantly, one of the basic criteria for a Fab Lab,^19^ which is also present in the Fab Lab Charter,^20^ is an open and free access to its facilities and tools at least part of the time each week. This often translates into an Open Day when anyone can come to visit or bring in or discuss a project, considered by most interviewees as an important moment to meet and attract new users. Besides Open Days, general assistance from staff, volunteers and other users is generally appreciated (Maldini 2013), which leads one of our interviewees to argue for the almost lack of barriers in contrast with other makerspaces. The Fab Lab network previews a series of other mechanisms, for instance optional, mandatory or targeted workshops, and also Mobile Fab Labs (Morel et al. 2015), to connect to potential users, citizens and communities, and also to other organizations for support, collaboration or partnerships, such as other makerspaces, associations such as repair cafés, museums, universities, art and design festivals, libraries, or local and national government bodies.

In maker cultures, a more active relationship with technology happens in the interplay of access to knowledge, tools and machines with collaboration and sharing with others. That is, such maker beliefs in openness, sharing and collaboration occur between formal mechanisms mostly related to open source principles, and more informal mechanisms mostly regarding community building as diversified as possible. The ultimate goal of engaging and building expanded communities who can use, create and share available knowledge, is translated into different practices in spaces and more spread-out networks such as Fab Labs, or in smaller organizations or communities such as OSHWA, under varying strategies and degrees of success at this stage.

### Addressing Social, Political and Ethical Points

Maker discourses and acts towards an active technological action, which break down deterministic relationships with technology and serve as an equalizing force, is infused with inclusive ideas of empowerment. Such empowerment, however, is still to be assessed within social, political and ethical conditions which may influence the actual access and use of knowledge, tools and technologies. In terms of access, what we call active technological action implies a degree of awareness about economic, social and cultural imbalances, arising for instance from gender, education, ethnicity, income or geographical origin. Such imbalances have gained attention in maker cultures more recently, creating a more complex understanding of who is a maker or in which context a maker is accessing and engaging in technological creation.

In a critical overview, it can be said that “despite powerful narratives of openness and individual empowerment (…) making in practice often falls short of these ideals. Many of the maker communities (…) are more exclusive in practice that their vision portrays (…)” (Ames et al. 2014). Within Maker Media realities, a 2012 study (MAKE/Intel 2012) based on a random sample of Maker Faire exhibitors and MAKE magazine and newsletter subscribers (solely residents in the US) showed that most of this maker community were male (81 %) college graduates (97 %) with median age of 44, owners of their own house or apartment (73 %) and a household income of $106,000, numbers very similar to a recent attendee study of Maker Faire Bay Area (Maker Faire 2014). Such a particular portrait in Maker Media has come under criticism, as analysed by Leah Buechley (2014), a scholar and practitioner in open hardware, in terms of white and male predominance in Make magazine’s covers (85 % male, 15 % female, 0 % non-white), editorial staff (87 % male, 13 % female and 0 % non-white) or article authorship, but also the type and higher cost of tools and kits being sold through their platforms.

Looking at the Fab Lab context, diversity and inclusion are acknowledged but still in an early stage of discussion and change. Perceptions from Fab Lab managers differ between those who hold that Fab Labs are mostly diverse in terms of gender, age or educational background, even comparing them to other makerspaces, and others expressing their awareness of a biased group of users, predominantly students, young, male and with an academic background. Diversity can also be equated as a matter of institutional affiliation or local context, or as a matter of promoted activities and focus, or even to geographical location, opening hours and fees, which may exclude people with low income, childcare responsibilities or with special working hours (Carstensen 2014). It echoes some debates in makerspaces, predominantly focusing in the gender divide, over the need to expand their activities and reserve space to areas such as arts/crafts, fashion, or textiles, the organization of girl subsections or groups inside makerspaces, or even to the creation of all-female or feminist hackerspaces like the Double Union in San Francisco.^21^ In recent years, many discussions (and disparate views^22^) are visible around discriminatory practices in hackerspaces towards women, queer, transsexual, and other minority groups, many times informally manifesting in members’ attitudes and stereotypes over abilities to make, learn or lead, but also in reported sexist and harassment incidents.^23^ But the main point here concerns a visible general concern towards the inclusion of women-in-tech or in “geek” communities, under-represented groups and economically-marginalized communities in maker communities. It can be argued that a more direct awareness is apparent at least in a part of the open knowledge and open source hardware movement, as seen in past and on-going discussions within these communities.^24^ In general, it is noteworthy the variety of initiatives now addressing for instance gender, economic disadvantages, learning gaps and ethnic diversity, like in non-profit organizations and/or programs as The Ada Initiative, Black Girls Code CoderDojo or Code.org.

It becomes clear that the prospects for makers to become active designers, creators and producers of technologies, goods and services, that is, to put into practice an open and critical intervention with our contemporary technical worlds, are framed within particular social, cultural and economic conditions. Such conditions can be at the very centre of their actions and developed into a critical and value-laden enactment in defence of more just, sustainable and democratic societies. Our empirical enquiries in some maker contexts looked more closely, for instance, at the existence of practices to elicit social transformation through making, that is, in what ways makers in these contexts are pursuing certain standards and values of common good, such as in the domains of civic action, sustainability, ethical codes of conduct and governance.

Orientations towards civic goals are at the core of OSHWA’s mission, definitely marking its character as a technology-oriented movement, when explicitly “encouraging the collaborative development of technology that serves education, environmental sustainability, and human welfare”.^25^ Some of the projects presented in the 2014 Open Hardware Summit organized by OSHWA specifically addressed or were oriented toward civic or public goals. We can underline here WikiHouse,^26^ which stands as an open source building system that openly shares design files for high-performance and low-energy homes to be customised, digitally manufactured and self-assembled by anyone; and the Open Source Beehives,^27^ started by a group of ecologists, beekeepers, makers, engineers and open source advocates and self-characterized as a network of citizen scientists, which makes openly available its hive, sensor kit designs and data to encourage everyone to build or purchase open source beehives.

Within the regular exhibitors in Maker Faire Rome, there was a variety of more DIY or more commercial projects dedicated to sustainability, or food and agriculture concerns, such as portable solar trackers to recharge portable devices,^28^ a solar oven and solar heating for housing,^29^ bio-climatic greenhouse systems, including containers for aquatic animal breeding and plant growing,^30^ or an automated aquaponic open source greenhouse for food production, adaptable to location and seasonal climatic conditions.^31^ Although it isn’t clearly a predominant strand in projects conducted in maker contexts such as the Maker Faire Rome, sustainability can still be considered as a recurrent topic of discussion in makerspaces, including their limitations for supporting it (Smith et al. 2013). Most of our Fab Lab interviewees mentioned a concern with more sustainable practices and in some cases users’ attention to resource management when they are, for instance, laser cutting an object using wood waste, or when they come to repair their own appliances or devices.^32^ On one hand, personal fabrication can be seen to encourage more sustainable production processes in terms of tailored consumption, resources, local origin of products or extended life cycles, but on the other hand, there are potential challenges in terms of dispersal of production capacity, diminished scale efficiencies, and intensified consumption through more personalized goods (Mota 2011).

When looking into makers alignment with normative stances which may frame critically their technological actions, attention to ethical choices in day-to-day activities or in the makerspaces’ governance models was addressed in our empirical incursions in Fab Labs. Above all, freedom to develop one’s own ideas or projects is privileged in such spaces, usually with no formal requirements or procedures beyond an initial contact and discussion with the Fab Lab managers. In membership-based Fab Labs that most of the times function with a 24/7 access, it is even more difficult to have direct knowledge of the projects being conducted in the Lab. None of the interviewees reported specific situations where they refused to host or to help with certain projects that they may have found inadequate or inappropriate. In their view, the openness of such spaces tends to act as informal control over the projects being developed.

Freedom and openness to act in contexts such as Fab Labs are seen to be at the core of their rationale and rules of operation, also in terms of funding models. In this respect, the formats for keeping up their material and financial existence, that is, their basic business model, are still being tested and not under prescribed or formal standards or codes. If it can be said that Fab Labs are privileged spaces for makers to start and develop open and collaborative projects (Menichinelli 2011), the actual sources or outputs of such activities are left to each user’s choice. The majority of our Fab Lab interviewees didn’t express any objection to private funding of Fab Labs, such as renting the space or prototyping services to companies, having company-funded Fab Labs, or receiving grants from larger corporations. Nevertheless, the Fab Lab Foundation generated a controversy in June 2014 by accepting a $10 million grant from Chevron Corporation to open up to 10 Fab Labs across the US in the next 3 years, located in areas where Chevron operates. Only one interviewee mentioned Chevron’s grant and expressed concern for the dangers of “fab-wash”, while advocating more discussion and transparency within the Fab Lab network about this type of situation.

One of the most visible controversies in maker cultures occurred when the MENTOR program developed by Dale Dougherty and Saul Griffith received a $10 million DARPA award, with the aim of bringing the practices of making into education and extend the maker movement into 1000 high schools over 3 years (O’Leary 2012). Mitch Altman, a renowned figure in the community and co-founder of San Francisco’s hackerspace Noisebridge, strongly expressed his disagreement to funding from military organizations, even for education purposes, and subsequently decided to leave the organization of Maker Faire (Altman 2012). From the diversity of situations here described, an active and critical technological action in maker cultures and spaces is now translated at different levels of awareness of existing social, economic, political and ethical conditions always framing our technological worlds, and even further, dissimilar postures which may or not lead to explicit oppositions to present imbalances and advocacy for social change.

## Concluding Remarks

Granted that makers can be connected to simpler DIY assemblies or to intricate hacker collectives, closer to technical dominations or more predisposed to community building, more interested in analogue experiments or in digital replications, makers cultures seen as a group are always based on a wide diversity of settings, territories, networks, people, artefacts and more importantly of different levels of awareness and commitment to a broader transformative power. Through snapshots of current maker cultures and some of their contexts of existence, we believe that an active technological action is mostly defined as a hands-on engagement with technology, that is, citizens creating, experimenting, producing and distributing alternative solutions on their own and together with others, locally and globally, or as part of local projects, as a response to their own everyday needs, or as the pursuit of new products and services, even if not always fully conscious of bigger pictures that involve changes in the significance of their own actions.

Pushing this argument further, maker cultures and their contexts can be seen as potential spheres of opposition to deterministic trends, that is, to develop fully its potential to turn users into active designers, producers, creators and distributors of knowledge, tools and machines. A makers’ relationship with technology needs to be understood as more complex than it usually is, not limited to individual assertions of freedom and creativity, but frequently connected to a set of social, economic, cultural, political and ethical conditions which vary according to the values and practices enacted by citizens, communities, networks and organizations. It is true that sustained changes towards active and critical interventions in maker cultures are more likely to be found in initiatives that are pursuing forms of technological action towards social change, which is visible for example in movements for open source or in projects for economic justice, gender equality and sustainability. But this has yet to compromise the transformation paths in themselves.

Whether or not a sizeable group of makers will turn in this direction will probably remain to be seen for a while, as a growing number of highly heterogeneous people is not only taking up this culture at a fast pace, supported by expanding communities and easier and cheaper access to tools and information, but also transforming it in ways no one could predict in the recent past, in terms of age, gender, ethnicity, abilities, needs, motivations, etc. What is a maker, what that person could be, or should be, will surely stand at the crossroad between diverse social, ethical and political understandings of technology and our relationships with it. But the notion of making as an increasingly significant technological action in itself, differentiated by the type of active and critical interventions in the smaller or larger technological sectors of our worlds, is becoming less and less controversial inside and outside maker worlds. This is now clearer and clearer in their inherent potential to break pre-set parameters of what is or not possible.

## Footnotes

1
http://www.whitehouse.gov/the-press-office/remarks-president-education-innovate-campaign.

2
http://www.whitehouse.gov/the-press-office/2014/06/17/presidential-proclamation-national-day-making-2014.

3
http://ec.europa.eu/archives/futurium/digital-agenda/en/content/future-horizon-2020-ri-challenges-and-opportunities.html.

4
http://cultura.gov.br/pontos-de-cultura1.

5
http://makerfaireafrica.com/.

6
http://www.fabfoundation.org/about-us/.

7
http://www.fabfoundation.org/fab-labs/what-is-a-fab-lab/.

8 For videos of presentations, https://www.youtube.com/channel/UCfQf3eKXX63TKPovdpUiZ1w.

9
http://www.makerfairerome.eu/project/smartize-it-low-cost-open-source-home-automation-139.

10
http://www.tamberg.org/iotzh/2014/makerfaire/.

11 See their Free Software Definition (What is free software?. Free Software Foundation). https://www.gnu.org/philosophy/free-sw.html).

12 See Debian Social Contract (Version 1.1 ratified on April 26 2004, https://www.debian.org/social_contract#guidelines; and also the Open Source Definition (http://opensource.org/osd) by the Open Source Initiative founded by Bruce Perens (also part of Debian) and Eric S. Raymond.

13 Stallman, R. Why Open Source misses the point of Free Software. https://www.gnu.org/philosophy/open-source-misses-the-point.html.

14
http://www.fabfoundation.org/fab-labs/fab-lab-criteria/.

15
http://fab.cba.mit.edu/about/charter/.

16 For videos of speakers and presentations, https://vimeo.com/album/3114662.

17
http://freedomdefined.org/OSHW.

18 See blog post and discussion on “The Failing of Fab Labs” http://fablab.nl/2013/09/29/the-failings-of-fablabs/.

19
http://www.fabfoundation.org/fab-labs/fab-lab-criteria/.

20
http://fab.cba.mit.edu/about/charter/.

21
https://modelviewculture.com/pieces/the-rise-of-feminist-hackerspaces-and-how-to-make-your-own.

22 Post by Liz Henry, February 3rd 2014, http://coloradomakerhub.org/2014/03/26/what-does-it-mean-to-be-a-woman-hackerspace-member/.

23 See Timeline of Incidents on the Geek Feminism blog, http://geekfeminism.wikia.com/wiki/Timeline_of_incidents.

24 See for instance the Topic Stream on “Gender and Diversity in Openness” in the 2012 Open Knowledge Festival, http://2012.okfestival.org/gender-and-diversity/.

25
http://www.oshwa.org/about/.

26
http://www.wikihouse.cc/.

27
http://www.opensourcebeehives.net/.

28
http://www.makerfairerome.eu/project/tracco-the-portable-solar-tracker-309/.

29
https://plus.google.com/117106484937664501696/posts.

30
http://www.solardomus.it.

31
http://plantsandmachines.de/.

32 Specifically addressing the topic of circular economy in repair cafés and hackerspaces (Charter and Keiller 2014), a recent survey acknowledged that, as expected volunteers at Repair Cafés are most strongly motivated to help others live more sustainably, to provide a valuable service to the community and to help improve product reparability and longevity, and instead, hackerspace members are more motivated to meet others with common interests, to be intellectually stimulated and to learn new skills.
